# Magnesium sulphate to prevent perioperative atrial fibrillation in cardiac surgery: a randomized clinical trial: A protocol description of the PeriOperative Magnesium Infusion to Prevent Atrial fibrillation Evaluated (POMPAE) trial

**DOI:** 10.1186/s13063-024-08368-3

**Published:** 2024-08-15

**Authors:** Manon Meerman, Marit Buijser, Lettie van den Berg, Anne-Marthe van den Heuvel, Gerard Hoohenkerk, Vincent van Driel, Luuk Munsterman, Roel de Vroege, Michael Bailey, Rinaldo Bellomo, Jeroen Ludikhuize

**Affiliations:** 1 Department of Intensive Care, HagaZiekenhuis, The Hague, The Netherlands; 2 Department of Cardiology, HagaZiekenhuis, The Hague, The Netherlands; 3 Department of Cardiothoracic Surgery, HagaZiekenhuis, The Hague, The Netherlands; 4 Department of Cardiac Anaesthesia, HagaZiekenhui, The Hague, The Netherlands; 5 Department of Perfusion, HagaZiekenhuis, The Hague, The Netherlands; 6 Department of Epidemiology and Preventive Medicine, Monash University, Melbourne, Australia; 7 Department of Intensive Care, Austin Health, Melbourne, Australia; 8 Australian and New Zealand Intensive Care Research Centre, Monash University, Melbourne, Australia; 9 Department of Critical Care, The University of Melbourne, Melbourne, Australia; 10 Data Analytics Research and Evaluation Centre, Austin Hospital, Melbourne, Australia; 11 Department of Intensive Care, Royal Melbourne Hospital, Melbourne, Australia

**Keywords:** Randomized clinical trial, Cardiac surgery, Postoperative atrial fibrillation, Magnesium sulphate

## Abstract

**Background:**

Postoperative atrial fibrillation (POAF) is a common and potentially serious complication post cardiac surgery. Hypomagnesaemia is common after cardiac surgery and recent evidence indicates that supplementation of magnesium may prevent POAF. We aim to investigate the effectiveness of continuous intravenous magnesium sulphate administration in the perioperative period to prevent POAF as compared to placebo.

**Methods:**

The (POMPAE) trial is a phase 2, single-center, double-blinded randomized superiority clinical study. It aims to assess the impact of perioperative continuous intravenous magnesium administration on the occurrence of cardiac surgery-related POAF. A total of 530 patients will be included. Eligible patients will be randomized in 1:1 ratio to the intervention or placebo group with stratification based on the presence of valvular surgery. The objective of the infusion is to maintain ionized magnesium levels between 1.5 and 2.0 mmol/L.

**Discussion:**

The primary outcome measure is the incidence of de novo POAF within the first 7 days following surgery, with censoring at hospital discharge. This trial may generate crucial evidence for the prevention of POAF and reduce clinical adverse events in patients following cardiac surgery.

**Trial registration:**

The POMPAE trial was registered at ClinicalTrials.gov under the following identifier NTC05669417, https://clinicaltrials.gov/ct2/show/NCT05669417. Registered on December 30, 2022.

**Protocol version:**

Version 3.3, dated 13–01-2023.

**Supplementary Information:**

The online version contains supplementary material available at 10.1186/s13063-024-08368-3.

## Background

Postoperative atrial fibrillation (POAF) is common after cardiothoracic surgery, affecting approximately 20–40% of patients [1, 2]. As such, POAF is associated with postoperative sequelae, including an elevated susceptibility to stroke, prolonged hospitalization and higher mortality rates [2–4].

Numerous pharmacological agents have undergone scrutiny as potential prophylactic interventions for POAF, including anti-arrhythmic drugs like amiodarone, beta-adrenergic receptor blockers and magnesium [5–8]. Regrettably, no prophylactic regimen has been conclusively established thus far.

Magnesium, a well-recognized and safe mineral, has garnered attention due to its potential to mitigate the risk of POAF. Hypomagnesaemia has been consistently linked to a greater risk of arrhythmias across diverse patient populations, including those undergoing cardiac surgery [9, 10]. The precise mechanistic underpinnings of the magnesium-arrhythmia interplay remain incompletely elucidated but may involve modulation of calcium channel function and the sodium–potassium membrane pump [11]. Magnesium supplementation, therefore, holds promise as an effective modality for attenuating early afterdepolarization-mediated triggered activity in the atria, thereby reducing the risk of POAF [2].

Several studies have demonstrated a reduction in arrhythmic incidents with magnesium supplementation, though these investigations are typically non-randomized and exhibit heterogeneity in dosing regimens and administration routes [11]. A recent pilot study adopting a serum magnesium target range of 1.5–2.0 mmol/L demonstrated a substantial diminution in POAF incidence, underscoring magnesium’s potential as a prophylactic intervention in cardiac surgery patient cohorts [8].

This manuscript describes the study protocol for the ‘PeriOperative Magnesium Infusion to Prevent Atrial Fibrillation Evaluated (POMPAE)’ trial, a double-blinded, placebo-controlled, randomized study. The primary research objective is to assess the efficacy of magnesium supplementation during the perioperative phase of cardiac surgery, with the aim of achieving targeted serum (ionized) magnesium levels within the range of 1.5 to 2.0 mmol/L.

## Methods

This study protocol has been developed following the recommendations of SPIRIT (Standard Protocol Items: Recommendations for Interventional Trials), a guideline for clinical trial protocols [12]. The SPIRIT checklist is shown in Supplementary Table 2.

### Trial design

The POMPAE trial is a randomized, single centre, double-blind placebo-controlled superiority trial conducted in the Haga Hospital in The Hague, The Netherlands. The aim of the trial is to analyse the potential of ionized magnesium levels in the serum between 1.5 and 2.0 mmol/L to prevent de novo atrial fibrillation in patients following cardiac surgery. For clarity, if magnesium levels are mentioned, they always indicate ionized magnesium levels.

Based on the protocol, upon initiation of anaesthesia, a continuous infusion of study medication (magnesium sulphate or Ringer’s lactate (placebo)) is initiated based on a magnesium level determined within the 72 h prior to surgery. If the preoperative level is below 1.0 mmol/L, the study infusion is preceded by a bolus of 10 mmol of magnesium to achieve stable magnesium levels in plasma. This study protocol (see ‘Trial intervention’ section) is based on previous pharmacokinetic studies performed in similar patient groups [13, 14].

### Setting and population

**Fig. 1:**
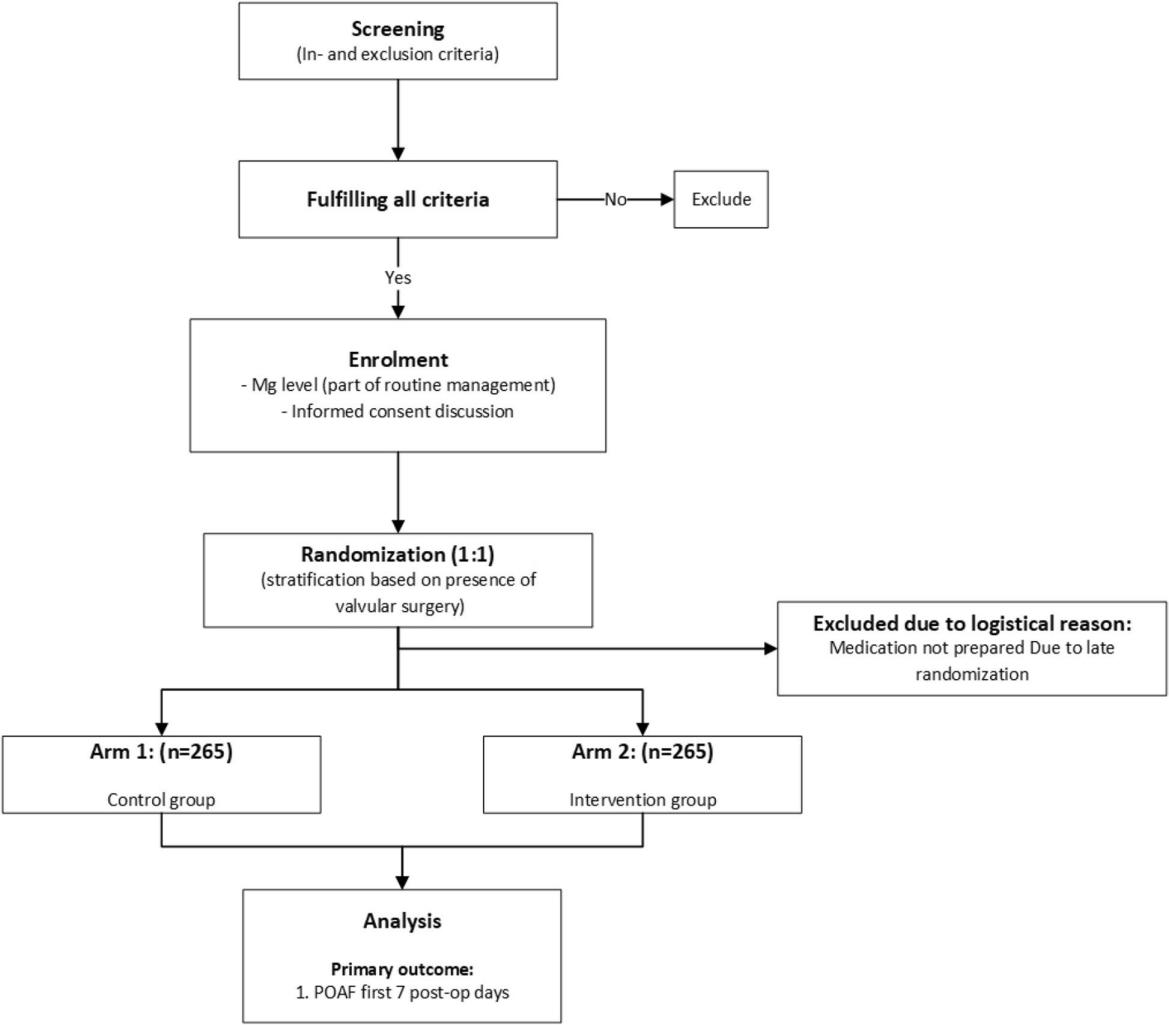
Study flow diagram

All patients 18 years or above undergoing semi-elective (indication for surgery is present 24 h or more prior to initiation of the surgery) cardiac surgery are eligible for inclusion in the trial. Table 1 provides a summary of both inclusion and exclusion criteria with the primary exclusion criterion being a past medical history of atrial fibrillation. Also, patients with severe renal impairment are excluded due to the risk of reduced clearance of magnesium and accompanying adverse events related to hypermagnesaemia. Both coronary artery bypass grafting (CABG) and/or valvular surgery are included with a 1:1 ratio with stratification according to each group being employed. The study flow diagram is shown in Fig. 1.

**Table 1:** Eligibility criteria for the POMPAE trial

Inclusion criteria
• Age ≥ 18 years
• Undergoing semi-elective CABG and/or valvular surgery
Exclusion criteria
• Previous history of AF or atrial flutter
• Concomitant rhythm-associated procedures (surgical ablation (MAZE) or pulmonary vein isolation)
• Pre-existent severe renal insufficiency (eGFR < 30 mL/min)

### Randomization and treatment masking (blinding)

All patients with an indication for cardiac surgery are screened (based on Table 1) for potential participation in the POMPAE trial. For elective patients, screening is performed at the preoperative clinic visit in the cardiac surgery department. For those patients being transferred from other hospitals, screening is performed at the time of arrival in the hospital. As only semi-elective surgeries are included, patients requiring immediate surgery, i.e. type A dissections or urgent CABG, due to main stem occlusion are excluded.

After information on the trial and trial documentation (see Supplementary Table 1), patients are either approached 1–2 h later (post admission to the hospital) for informed consent or, for those with elective surgery, directly upon admission. After inclusion, randomization is performed in Castor [15] (web-based randomization and database application) by the cardiac surgery department, see later for more details.

As part of admission routine, a magnesium level is obtained from regular blood works. The first study intervention is the administration of study medication post induction of anaesthesia. The pharmacy department receives an email post randomization with treatment allocation and prepares the trial drug. The team responsible for patient care (in the operating room (OR), intensive care unit (ICU) and later, the cardiac surgery department) remains fully blinded to treatment allocation. The study drug is labelled with the patient’s information and transported to the OR prior to the beginning of surgery. Since both MgSO_4_ and the placebo (Ringer’s lactate) are clear fluids, treatment allocation remains concealed to the anaesthesiologist and ICU staff treating the patient postoperatively. Serum magnesium levels are measured according to protocol (see ‘Trial intervention’ section). The trial participants and the study team remain blinded to treatment allocation during the conduction of the trial.

However, as magnesium levels are measured during the study as part of usual care, staff may suspect allocation. As atrial fibrillation is objectively assessed by ECG readings and all other procedures (including rescue medication (anti-arrhythmic drugs) and procedures (electro cardioversion)) are monitored, the chance of influence of this awareness on the outcome parameters is deemed low. Unblinding of a patient is possible as per clinical directives of the treating clinical team. The principal investigator and/or at least one person from the study team is available 24/7. This person will then reach the contact person within the pharmacy department who is able to unblind the patient and the study team will relay this to the treating physician. Unblinding will be documented in the appropriate manner within the trial documentation.

### Trial intervention

**Fig. 2:**
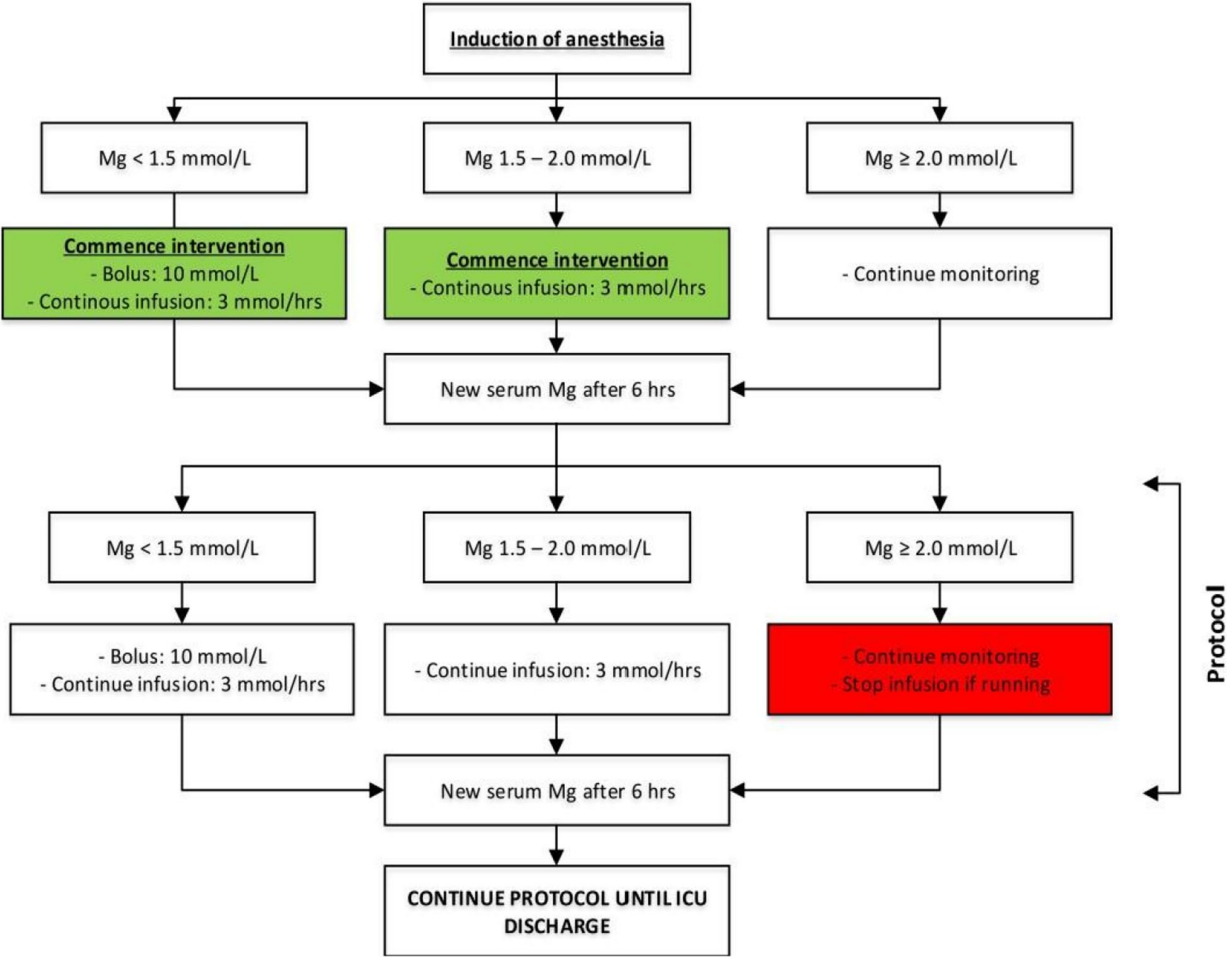
Study protocol for administration of study medication (bolus and/or continuous infusion). Legend: This flowchart shows the study protocol for the measurement of (ionized) magnesium levels and administration of study medication. Patient randomization is not shown within the figure. This event occurs as part of the first step after which a magnesium serum level (part of regular clinical routine) is determined. This level (maximum of 72 h before start of surgery/induction of anaesthesia) determines the procedures undertaken post induction of anaesthesia. (See ‘Trial intervention’ for more details). If magnesium levels fall below 1.0 mmol/L, an unlabelled bolus of 10 mmol magnesium sulphate is administered to the patient as part of regular clinical practice within the hospital for patients undergoing cardiac surgery

Figure 2 shows the study protocol from randomization to ICU discharge, after which time all trial medication and magnesium level measurements (based on the study protocol) are ceased.

Based on the magnesium level taken prior to surgery (maximum of 72 h before surgery), the anaesthesiologist will initiate the study medication after induction of anaesthesia. If magnesium level is below 1.0 mmol/L, a bolus of magnesium is provided, and the continuous infusion is initiated as soon as the central line is in situ.

In general, the aim of the study is to achieve stable magnesium level between 1.5 and 2.0 mmol/L within the intervention group. If the magnesium levels fall below 1.5 mmol/L, a bolus of 10 mmol magnesium sulphate will be given. The continuous infusion is initiated or continued based on the protocol with a trail drug administration rate of 3.0 mmol per hour. In case magnesium levels are above 2.0 mmol/L, the trial medication is (temporarily) ceased. Magnesium levels are measured each 6 h and the study medication is adjusted based on its level as described study protocol.

After discharge from ICU, the study medication in ceased. In the case of readmission to ICU, no trial medication will be initiated as the trial only focuses on the direct perioperative course of a patient during the index operation and the post-surgery ICU admission.

As part of regular clinical practice within the Haga Hospital for patients undergoing cardiac surgery, the following protocol is followed in the OR and ICU. In the OR, before liberation from the cardiopulmonary bypass (CPB), a slow bolus of 2 mg of milrinone, 10 mmol of magnesium sulphate and 4.5 mmol of calcium gluconate is administered to all patients. Within the entire perioperative period (including the ICU), if magnesium levels are below 1.0 mmol/L, administration of 10 mmol magnesium is always provided. Specifically, for the period within the OR, a magnesium level is determined 30 min post induction of anaesthesia. In accordance with hospital practice, only if the level is below 1.0 mmol/L, unlabelled magnesium is administered prior to liberation from the CPB. Information on all these procedures and administrations is collected.

### Concomitant therapies

Within the POMPAE trial, there are no restrictions to using concomitant therapies to treat arrhythmias occurring in the OR, ICU or on the ward. The use of any medication to treat (supra ventricular) arrhythmias such as beta blockers or anti-arrhythmic drugs (e.g. amiodarone, sotalol, lidocaine) will be recorded in the database and will be left at the discretion of the treating physician.

### Outcomes

The primary aim of the POMPAE trial is to investigate the effects of magnesium sulphate on the prevention of post cardiac surgery atrial fibrillation. The primary outcome is the incidence of postoperative atrial fibrillation (POAF) in the first 7 postoperative days (censored at hospital discharge) consisting of newly diagnosed AF over a period of 5 min or longer by ECG recording. The secondary study parameters/endpoints are AF during the 28 days post-surgery, the duration of POAF and peak heart rate recorded, hospital and ICU lengths of stay, the duration of mechanical ventilation and duration of inotropic or vasopressor support. Also, the combined outcome including 28-day post-surgery mortality, stroke, pulmonary embolism, delirium (requiring any form and/or duration of anti-psychotic medication) and infection requiring antibiotics will be measured.

### Data collection and management

All collected data will be managed in an online database (Castor EDC). The handling of personal data will comply with the EU General Data Protection Regulation and the Dutch Act on Implementation of the General Data Protection Regulation. The collection and processing of participants’ personal information will be done in such a way that only the data necessary to answer the research questions listed in this protocol are collected and stored. All involved subjects will receive a code when inclusion in Castor is recorded. This code is not based on the patient initials and/or birth date. Per site a subject identification code list is used to link the data to the subject. Only the investigators involved in the study will have access to this subject identification list. All data will be treated confidentially. In Castor EDC, no patient’s names or dates of birth will be recorded.

Research team members will collect data from the electronic patient records. Baseline data such as gender, previous medical history and medication use will be collected. ECGs will be performed every 6 h during the period of study medication administration. After discharge from the ICU, daily ECGs will be performed until the seventh postoperative day or unless discharge from the hospital occurs earlier. Additional ECGs can be performed upon the discretion of the clinician and are also recorded within the study database. All ECGs performed within the first 28 days postoperatively will be collected, including those performed in the referring hospitals (for instance if patients are transferred post-surgery before being eligible for home discharge). All other variables are collected from the patient data management systems and transferred directly into the electronic Castor database. A full list of all collected data variables can be found in Table 2.

**Table 2:** Data to be collected in the POMPAE trial

Time of collection of type of data	Data collected
Screening	• Patient’s initials and date screened • Patient’s type of surgery (CABG and/or valvular surgery) • Informed consent obtained yes/no
Baseline (if informed consent obtained)	• Gender, height and weight, EuroScore II • Previous medical history including previous CVA (i/h), MI, PCI, cardiac surgery • Comorbidities including hypertension, dyslipidaemia, asthma/COPD, chronic renal failure, diabetes mellitus, thyroid disease and OSAS • History of smoking including pack years • Left ventricular ejection fraction (%) on preoperative echo
Perioperative data	• Date and time of hospital admission, serum Mg, ECG (rhythm, rate, conduction intervals) • Date and time of initiation of anaesthesia, intubation, start and end of surgery • Date and time of initiation of bolus study medication and continuous infusion including Mg level post induction • Duration of aortic clamp, CPB, type and amount of cardioplegia used • Rescue therapies during surgery (use of medication including circulatory support, assist devices, pacemakers, etc.)
Intensive care	• Date and time of admission/discharge to ICU, extubation, use of circulatory support (type, dosage and duration) and discharge including destination • Mg and ECG data including date and time stamp and alterations to the study medication • Use of antiarrhythmic medications including beta blockers, Mg (unlabelled) amiodarone, etc • Procedures including re-surgery, pacing (in- and external)
Ward/CCU	• Date and time of admission/discharge to the ward/CCU, use of circulatory support (type, dosage and duration) • Continue ECG daily • Use of antiarrhythmic medications including beta blockers, Mg (unlabelled) amiodarone, etc • Procedures including re-surgery, pacing (in- and external)
Outcome and assessment data	• Outcome of the admission (discharged alive, lost to follow-up or diseased) • Other complications including pulmonary embolism, stroke, delirium (required usage of newly described anti-psychotics) or infections requiring antibiotics
Adverse events	• Adverse event description, timing, causality and resolution until discharge • The same was employed for serious adverse events and SUSAR
Protocol deviations	• Occurrence, description including date and time • Exclusion based on logistics (medication does not present at time of surgery, Mg level expired or other with description) • Stopping rule encountered, description including date and time

ECGs will be evaluated by the Cardiology team. For patients with a single ECG displaying AF, an electrophysiologist will reevaluate all ECGs performed in this patient. If a discrepancy occurs between these two evaluators, a third (also electrophysiologist) will make the final evaluation. As part of Good Clinical Practice Guidelines (GCP), the local monitoring board will conduct regular monitoring visits (3–6 months) to ensure high-quality data output and good conduct of the study. Although the monitoring personnel are part of the study hospital staff, they are independent from the research department and investigators.

### Ethics and good clinical practice

The trial was approved by the medical ethics committee (METC) Leiden-Den Haag-Delft. The trial was registered at ClinicalTrials.gov (NTC05669417). All patients must be able to provide informed consent before initiation into the study. The trial is to be conducted according to the standard requirements of Good Clinical Practice [16].

In case of modifications within the protocol, the principal investigator is responsible for the communication to all relevant parties.

### Data safety monitoring committee and interim analysis

The data safety monitoring board (DSMB) consists of professor P. Nanayakkara (MD, PhD and Consultant Acute Internal Medicine, VU Medical Centre Amsterdam), assistant professor H. Merten (PhD methodologist/Statistician, VU Medical Centre Amsterdam) and J. Janson (MD and consultant Intensive Care, Leiden University Medical Centre). The DSMB convenes after the first 10 patients, after 6 and 12 months and annually if required.

The role of the DSMB is to ensure that the rights and safety of the study participants are protected. The DSMB has been enabled to review and approve the study protocol. During the planned meetings, they will evaluate all reported suspected unexpected serious adverse events as they occur. A continuously updated summary of protocol deviations and adverse events will also be provided to the DSMB.

A formal interim analysis will be performed after the 265th participant (50%) has completed the surgery. The DSMB together with the trial statistician will be required to inform the POMPAE trial team of any evidence relevant to continuing the trial and recommendations on the further conduct of the trial. The DSMB acts in an advisory capacity to the POMPAE trial team, who are ultimately responsible for the conduct of the trial.

### Adverse events and participant stopping rules

All patients eligible for inclusion in the POMPAE trial will receive information regarding the trial and will be provided with the required written information including the informed consent form. All participants are expected to sign the informed consent before the initiation of study medication in the presence of one of the doctors from the cardiothoracic surgery department. Participants can withdraw from the trial at any moment and for any reason.

As magnesium is a well-known compound and used (off-label) to treat arrhythmias, it is considered a safe treatment option. As there is a change of hypermagnesaemia with potential cardiac arrhythmias (AV-block) and neurological symptoms (including (partial) paralysis), the trial medication will only be provided in highly monitored areas such as the OR and the ICU. Therefore, the associated risks with trial participation are considered low. However, adverse events may still occur and will be reported according to the established practice in line with GCP conducted clinical trials [17].

Table 3 shows the list of (serious) adverse events as determined within the POMPAE trial. Importantly, tamponade and/or serious bleeding requiring potential re-surgery have been excluded from the analysis because these are considered not specific to magnesium treatment [18]. The investigator reports all (S)AEs to the sponsor without delay, after which the sponsor will report the SAEs to the accredited METC that approved the protocol.

**Table 3:** (Serious) adverse events and stopping rules

Adverse events:
• Serum magnesium levels above 3 mmol/L
• Requirement of insertion of temporary pacemaker wires (trans-jugular, transfemoral)
• Requirement for external pacing
Serious adverse events:
A serious adverse event is any untoward medical occurrence or effect that:
• Results in death
• Is life threatening (at the time of the event)
• Requires hospitalization or prolongation of existing inpatients’ hospitalization
• Results in persistent or significant disability or incapacity
• Is a congenital anomaly or birth defect
• Any other important medical event that did not result in any of the outcomes listed above due to medical or surgical intervention but could have been based upon appropriate judgement by the investigator
Stopping rules:
• Development of postoperative oliguria (< 200 mL in previous 6 h) and/or rise in creatinine with resulting eGFR of < 30 mL/min
• Development or presence of significant hypotension (irrespective of cause) persisting for > 1 h with the requirement of norepinephrine support of > 0.2 mcg/kg/min
• Presence or development of third-degree heart block

In relation to the POMPAE study, participant stopping rules have been formulated as depicted in Table 3. If a stopping rule occurs, study medication is permanently ceased with the continuation of ECG measurements as per protocol.

### Sample size and power

With 500 patients, this study will have 80% power (2-sided *p* value of 0.05) to detect a relative reduction of 40% (absolute reduction 25% vs 15%) in the incidence of POAF. To allow for consent refusal or loss to follow-up, 530 patients will be recruited.

### Statistical analysis plan

All analysis will be performed based on modified intention-to-treat principles, excluding only patients that withdrew consent or were unable to receive study medication due to logistical reasons. All eligible patients consenting to participate will be screened and randomized to maximize inclusion. However, as study medication could not be prepared by the pharmacy department outside of regular working hours, patients not able to receive medication at the start of anaesthesia, despite consent, will be excluded from analysis.

All data will initially be assessed for normality. Baseline imbalance will be determined using chi-square tests for equal proportion, Student tests for normally distributed data and Wilcoxon rank sum test for non-parametric data with results presented and *n* (%), mean (standard deviation) or median (interquartile range) respectively.

The primary outcome (POAF) and other binomial secondary outcomes will be analysed using generalized modelling (accounting for stratification variables of surgery type) with relative risk (95% CI) determined using a log binomial model. A risk difference (95% CI) determined using a binomial distribution with an identify link and odds ratios (95% CI) using a logistic binomial model will also be employed, where appropriate. Moreover, it is prescribed that sensitivity analysis will be performed adjusting for baseline imbalance (*p* < 0.2) and baseline covariates previously identified as being known to be linked to outcome (age, left ventricular function, type and length of surgery including time on CPB, use of vasopressors and inotropes). Where there is missing data for the primary outcome, multiple imputation will be employed.

To account for the competing risk of death, duration outcomes (time to POAF, lengths of stay, ventilation, inotropic or vasopressor support) will be analysed using competing risk regression with results reported as hazard ratios (95% CI) and presented as cumulative incidence curves. All analysis will be performed using SAS version 9.4 (SAS Institute Inc., Cary, NC, USA) and a two-sided *p* value of 0.05 will be used to indicate statistical significance. For the analysis of the secondary endpoints, adjustment for multiplicity will be performed using the Holm-Bonferroni method to reduce changes of type 1 error.

The protocol requires that an independent statistician will perform the interim analysis when the halfway mark of patient recruitment is achieved (*n* = 265). Based on a discussion between the statistician and the DSMB members as part of a closed session, the protocol requires that the DSMB will recommend one of the following:

- The trial should cease prematurely based on efficacy
- The trial should cease prematurely based on futility
- The trial should end prematurely (or be monitored more closely) for safety reasons
- The trial should continue until completion with or without modification

Final decision on whether to prematurely stop the trial will be performed by the study committee.

### Presentation of outcome data

Our proposed tables and figures for the primary manuscript describing the outcomes of the POMPAE trial are listed in Table 4.

**Table 4:** Planned tables and figures

Proposed table and figures for the main manuscript
• Table 1: Baseline characteristics
• Table 2: Primary and secondary outcomes
• Fig. 1: Consort diagram showing assessment of patient eligibility, random assignment of patients, analysis population and flow of patients in the POMPAE trial
• Fig. 2: Ionized magnesium levels in treatment and placebo group
• Table 3: Compliance to POMPAE medication delivery protocol
Proposed table and figures for the supplementary appendix of the main manuscript
• Table S1: Additional baseline characteristics
• Table S2: Protocol deviations and intervention altered compared to protocol
• Table S3: Rescue therapies employed during trial
• Table S4: (Serious) adverse events and stopping rules applied
• Table S5: Analysis of primary and secondary outcomes between stratification groups
• Table S6: Unadjusted analyses in the intention-to-treat population
• Figure S1: Mean, lowest and highest Mg level during intervention period by treatment group
• Figure S2: Time-weighted mean ionized Mg level during intervention period by treatment group

## Discussion

Our trial aims to reduce the incidence of postoperative atrial fibrillation by administration of magnesium sulphate to achieve stable ionized magnesium levels in the serum between 1.5 and 2.0 mmol/L.

After careful pharmacokinetic investigations and a before-after trial employing a protocol very similar to that of the POMPAE trial, this is the first large, randomized trial investigating the potential of magnesium to prevent POAF. As magnesium has the potential for side-effects (cardiac rhythm alterations including heart block, paralysis and neurological sequelae including coma), administration of magnesium is limited to tightly controlled areas (OR and ICU). As our trial is conducted in a large peripheral hospital with a high turn-over of low to intermediate risk cardiac surgery and has limitations related to acute (high risk) surgery, there is the potential for selection bias and reduced external validity.

Due to the nature of cardiac surgery and short time frames between indication for cardiac surgery and eventual surgery itself, some patients may not be able to receive study medication as the pharmacy department may not be able prepare this in time. This may lead to a degree of attrition bias. However, only low numbers of patients are expected to be removed from the study in this way. Therefore, the analysis of the POMPAE is designed as a modified intention to treat analysis.

In conclusion, the POMPAE trial is a single-centre RCT which is designed to evaluate the effects of continuous magnesium sulphate infusion for the prevention of POAF in patients undergoing cardiothoracic surgery. The primary outcome is the occurrence of POAF in the first 7 days after cardiac surgery. Secondary outcomes include the occurrence of POAF in the first 28 postoperative days, length of hospital and ICU stay and duration of mechanical ventilation and vasopressor and/or inotropic support.

## Trial status

The protocol version number is 3.3 dated January 13, 2023. The ethics committee approved this protocol dated March 9, 2023. Currently, the POMPAE is recruiting although on pause as we are awaiting the results of the interim analysis.

### Supplementary Information

Additional file 1: SPIRIT checklist.

Additional file 2: Supplementary Table 1 Informed consent documents (translated from Dutch).

Additional file 3: Supplementary Table 2 SPIRIT figure.

## Data Availability

Data is available on request from the corresponding author. JL, RB and MB will have full access to the data to final data set including the post-trial period. Dissemination of information from the trial results will be performed in peer reviewed manuscripts and for those with general interest (including patients), in the magazine of the hospital.
