# Supplementary material for: Magnesium sulphate to prevent perioperative atrial fibrillation in cardiac surgery: a randomized clinical trial: A protocol description of the PeriOperative Magnesium Infusion to Prevent Atrial fibrillation Evaluated (POMPAE) trial
Source: Trials. 2024 Aug 15;25:540. doi: 10.1186/s13063-024-08368-3 (PMC11328354; doi:10.1186/s13063-024-08368-3)
Supplement: Supplementary file 2 — Additional file 2: Supplementary Table 1 Informed consent documents (translated from Dutch). [file 13063_2024_8368_MOESM2_ESM.docx]

**Supplementary table 1. Informed consent documents (translated from Dutch)**

**Subject Information for Participation in Medical Scientific Research**

**Administration of magnesium sulfate to prevent atrial fibrillation around heart surgery**

*Official Title: POMPAE trial. Peri-Operative Magnesium infusion to Prevent Atrial fibrillation Evaluated.*

**Introduction**

Dear Sir/Madam,

With this information letter, we would like to ask you to participate in medical scientific research. Participation is voluntary. You are receiving this letter because you are undergoing heart surgery soon. You will read about the type of research, what it means for you, and its benefits and drawbacks. It's a lot of information. Would you please read through it and decide if you want to participate? If you choose to participate, you can fill out the form found in Annex C.

**Ask Your Questions**

You can make your decision based on the information in this information letter. Additionally, we recommend the following:

- Ask questions to the researcher providing you with this information.
- Discuss this research with your partner, family, or friends.
- Ask questions to the independent expert, Dr. Vriend (cardiologist).
- Read the information on [www.rijksoverheid.nl/mensenonderzoek](http://www.rijksoverheid.nl/mensenonderzoek).

**1. General Information**

The HagaZiekenhuis has initiated this research. Below, we refer to the HagaZiekenhuis as the 'sponsor'. Researchers may include doctors, investigators, and research nurses. They conduct the research at the HagaZiekenhuis.

This research requires 530 participants. The medical-ethical review committee LDD (Leiden-Den Haag-Delft) has approved this research.

**2. What is the purpose of the research?**

In this research, we are examining how well magnesium sulfate works to prevent atrial fibrillation after open-heart surgery. We are comparing the effectiveness of preventing atrial fibrillation using magnesium sulfate with the effectiveness of a placebo. A placebo is a substance without an active ingredient, a 'dummy' substance.

**3. What is the background of the research?**

Atrial fibrillation is a heart rhythm disorder that often occurs in the elderly or people with heart disease. Additionally, this condition often occurs in people who have recently undergone heart surgery. Atrial fibrillation causes the heart to beat irregularly, and usually, attempts are made to restore regular heart rhythm with medications. Magnesium is a mineral naturally found in our diet and thus in the human body. Intravenous administration of magnesium (in the form of magnesium sulfate) in the hospital is normally used to restore regular heart rhythm when atrial fibrillation is present. Research also shows that administering magnesium to patients who have just undergone heart surgery reduces the risk of developing atrial fibrillation shortly after heart surgery. However, the studies conducted so far do not meet the highest standard of quality. To meet this standard, a comparison is needed with a group of patients who are simultaneously treated with a placebo. With this research, we aim to make this comparison. If successful, magnesium sulfate could potentially be used in the future as part of the standard treatment around heart surgery.

**4. How does the research proceed?**

*How long does the research last?*

If you participate in the research, it will last approximately 1 week in total. The examinations take place during your hospitalization in connection with your heart surgery.

*Step 1: Are you suitable to participate?*

We first want to know if you are eligible to participate. Therefore, the researcher conducts several examinations:

- Physical examination. For example, the researcher listens to your heart and lungs, measures your blood pressure and heart rate.
- Blood tests. The researcher takes a blood sample from you to test for kidney function disorders. The kidneys are responsible for excreting magnesium through urine. In case of severe kidney dysfunction, you are not suitable to participate in the research because there is a greater risk of high magnesium levels in the blood. We will inform you if you have kidney dysfunction, and if this was not previously known, we will notify your general practitioner for additional tests and treatment.
- Electrocardiogram (ECG).
- Examination of your medical history. If you are already known to have atrial fibrillation or severe kidney dysfunction, you cannot participate in the study.

The above examinations are already part of the standard procedure around heart surgery. So, you do not need to undergo any additional examinations or come to the hospital separately for this research.

*Step 2: The treatment:*

We will treat you with magnesium sulfate for 1 to several days after the heart surgery, as long as you stay in the intensive care unit.

For this research, we create 2 groups:

- Group 1. People in this group receive magnesium sulfate administered intravenously.
- Group 2. People in this group receive a placebo administered intravenously unless the magnesium level in your blood reaches a very low level. In that case, magnesium sulfate is administered until the magnesium level in your blood returns to a normal level. This is already done as standard for all patients.

A lottery determines which treatment you receive, with a 50% chance of receiving the study drug. You and the researcher do not know which group you are in. If it is important for your health, this information can be looked up.

*Step 3: Examinations and measurements*

Before your heart surgery, you will be admitted to the hospital, at least 1 day before the surgery. During your hospitalization, the following examinations are conducted:

- Electrocardiogram (ECG).
- Blood collection: to determine the magnesium level in your blood on the day before the surgery as part of routine blood collection.
- Holter monitoring: to monitor your heart rhythm on the nursing ward.
- During your stay in the intensive care unit, magnesium levels in your blood will be determined every 6 hours. This does not require additional blood sampling because we can take it from one of the infusions you have already received before the surgery.

*Step 4: Follow-up*

There are no specific follow-up appointments required for the study. During the period of your hospitalization, an ECG will be performed daily. The same applies if you unexpectedly need to be readmitted within the 28-day period after the surgery due to a reason discussed with the cardiologist/cardiac surgeon.

So, you do not need to come to the hospital additionally if you decide to participate in the POMPAE study.

*"What is different from regular care?*

As described above, magnesium sulfate is or is not administered, and extra checks of the magnesium level in your blood will take place during your admission to the intensive care unit (see step 3 above). Additionally, during the 7 days after your surgery, a daily electrocardiogram (ECG) will be performed. This will happen 2 times more frequently than if you were not participating in the study. These extra ECG checks will occur on days 5 and 7 unless you are discharged home during this period. Then, these extra ECG checks will be omitted.

In the period after these 7 days, there is generally no further need for follow-up. Unless a situation arises as described immediately below this paragraph. Then, only the sharing of the information below will occur, and no additional tests.

In addition, there is nothing different from regular care during the study.

**5. What agreements do we make with you?**

We want the research to go smoothly. That's why we make the following agreements with you:

- You'll get in touch with the researcher in the following situations if they occur within the 28 days after the surgery:
  - You want to start using other medications. Even if they're homeopathic remedies, natural remedies, vitamins, or medications from the drugstore;
  - You're admitted to a hospital or treated there;
  - You suddenly have health problems;
  - You no longer want to participate in the research;
  - Your phone number, address, or email address changes.

**6. What side effects, adverse effects, or discomfort might you experience?**

The treatment being studied, administering magnesium sulfate around your heart surgery, may cause side effects or adverse effects. The following side effects can occur:

- Flushing (feeling of warmth/blushing);
- Drowsiness/fatigue.

The following side effects are rare but can be serious:

- A lowered heart rate and rhythm disturbances;
- Low blood pressure;
- Muscle weakness.

Such a side effect is very rare and is only known to occur at very high levels of magnesium in the blood, much higher than those targeted in the study. Because magnesium administration only occurs during surgery and in the intensive care unit, you'll be continuously monitored. Your heart rate, blood pressure, and consciousness (once you're out of anesthesia) will be constantly checked.

If the magnesium level in your blood becomes too high, the magnesium infusion will be stopped immediately. If you still show signs of serious side effects, you will be treated for them promptly. The mentioned side effects are usually treatable, often by providing extra fluids or a diuretic.

The therapy being investigated may also have side effects that we are not yet aware of. Since the medication has been used for a long time for various other reasons (including in gynecology for preeclampsia), the likelihood of this is very small.

**7. What are the advantages and disadvantages of participating in the research?**

Participating in the research can have both advantages and disadvantages. Below, we list them. Think carefully about this and discuss it with others.

When you are assigned to the group of patients receiving magnesium sulfate, this may reduce the risk of you developing atrial fibrillation after the surgery, but this is not certain. So, participating in the research does not mean you receive better care. Since atrial fibrillation after surgery is common (even with potential participation), you will receive treatment as the cardiologist normally does. This may involve taking additional medications such as blood thinners and other medication for heart rhythm, which are no longer part of the study.

Participating in the research may also have disadvantages, especially potential side effects as described in paragraph 6. Additionally, some extra tests are performed (ECG), which represents a limited extra burden for you.

Participation in research is voluntary, and we hope to improve the treatment for atrial fibrillation after heart surgery through the POMPAE study.

*Do you not want to participate?*

You decide whether to participate in the research. If you choose not to participate, you will receive standard treatment after heart surgery (no extra magnesium per infusion, unless the value in your blood is low). Your doctor can tell you more about the treatment options available and their pros and cons.

**8. When does the research stop?**

The researcher will inform you if there is new information about the research that is important for you. The researcher will then ask you if you want to continue participating.

For you, the research stops in these situations:

- All examinations according to the schedule are completed.
- You want to stop participating in the study yourself. You can do this at any time. Just inform the researcher immediately. You don't have to explain why you're stopping. You'll then resume receiving standard treatment after heart surgery. For your safety, the researcher may schedule one or more follow-up checks.
- The researcher deems it advisable for you to withdraw.
- One of the following entities decides to terminate the study:
  - HagaZiekenhuis
  - Government authorities
  - The medical ethics committee overseeing the research.

*What happens if you stop participating in the study?*

The researchers will use the data collected up to the point of your withdrawal. The entire study concludes once all participants have completed it.

**9. What happens after the study?**

*Can you continue to use the medications?*

The medications used in this study are exclusively administered in the operating room and the intensive care unit, where you will be during and immediately after heart surgery.

*Will you receive the results of the study?*

A summary of the research results will be made available to the patients.

**10. What do we do with your data?**

If you participate in the study, you also consent to the collection, use, and storage of your data.

*What data do we store?*

We store the following data:

- Your name
- Your gender
- Your date of birth
- Health-related data
- (Medical) data collected during the study

*How do we protect your privacy?*

To protect your privacy, we assign a code to your data. Only this code is used on all your data. The key to the code is kept securely in the hospital. When processing your data, we only use this code. Reports and publications about the study do not reveal your identity.

*Who can access your data?*

Some individuals may access your name and other personal information without the code. These are people who ensure that the researchers are conducting the study properly and reliably. These individuals include:

- Members of the committee overseeing the safety of the research.
- A monitor working for the researcher/sponsor.
- National and international regulatory authorities, such as the Health and Youth Care Inspectorate (IGJ).

These individuals keep your data confidential. We ask for your permission for this access.

*How long do we keep your data and bodily material?*

We retain your data for 25 years at the HagaZiekenhuis.

*Can we use your data for other research?*

Your data may still be relevant for other scientific research on your condition and/or treatment method after this study concludes. This could include research on other heart rhythm disorders such as atrial fibrillation. In the consent form, you indicate whether you agree to this. If you do not give consent, you can still participate in this study.

*What happens with unexpected findings?*

During the study, we may accidentally discover something important for your health or the health of your family members. The researcher will then contact your general practitioner and/or treating specialist. You will then discuss with your general practitioner or specialist what needs to be done. You provide consent with the form for informing your general practitioner or specialist.

Can you revoke your consent for the use of your data?

You can revoke your consent for the use of your data at any time. This applies to both this study and other research. But please note: if you revoke your consent and researchers have already collected data for a study, they may still use this data.

*Would you like to know more about your privacy?*

- If you want to know more about your rights regarding the processing of personal data, visit www.autoriteitpersoonsgegevens.nl.
- Do you have questions about your rights? Or do you have a complaint about the processing of your personal data? Then contact the person responsible for the processing of your personal data. For your study, this is:
  - HagaZiekenhuis. See Appendix A for contact details.
- If you have complaints about the processing of your personal data, we recommend that you first discuss this with the research team. You can also contact the privacy officer of your hospital or the Data Protection Authority.

*Where can you find more information about the research?*

You can find more information about the research on the following website: https://clinicaltrials.gov/ct2/home. The POMPAE study is registered under number NCT05669417 with the direct link: https://clinicaltrials.gov/ct2/show/NCT05669417. After the study, the website may provide a summary of the results of this research. You can find the study by searching for the study number on the aforementioned website.

**11. Will you receive compensation for participating in the research?**

Participation in the research does not cost you anything. You will also not receive compensation for participating in this research.

**12. Are you insured during the research?**

Insurance has been taken out for everyone participating in this research. The insurance covers damages caused by the research. However, not all damages are covered. More information about the insurance and exceptions can be found in **Appendix B**. It also indicates to whom you can report damages.

**13. We inform your treating specialist and pharmacist**

The researcher will send a letter to your treating specialist to inform them that you are participating in the research. This is for your own safety. In case of uncertainties, we can contact your (family) doctor, treating specialist, or pharmacist, for example, regarding your medical history or the medications you use.

**14. Do you have any questions?**

You can ask questions about the research to the research team and/or the doctor present at the preoperative consultation. Would you like advice from someone who has no vested interest? Then go to Dr. Vriend. He knows a lot about the research but is not involved in it.

Do you have a complaint? Discuss this with the researcher or the doctor treating you. Would you prefer not to? Then go to the complaints officer of your hospital or the Data Protection Authority. Appendix A contains where you can find them.

**15. How do you give consent for the research?**

You can take your time to consider this research. Then, you inform the researcher whether you understand the information and whether you want to participate. Do you want to participate? Then fill in the consent form that you find with this information letter. You and the researcher will both receive a signed version of this consent declaration.

Thank you for your time.

**16. Appendices to this information**

A. Contact details

B. Information about the insurance

C. Consent form for research participants

**Appendix A: Contact Information for HagaZiekenhuis**

Principal Investigator: Dr. Ludikhuize, Intensivist at HagaZiekenhuis

Accessible via CardioResearch or the Intensive Care department

CardioResearch HagaZiekenhuis

Phone number: +31 70 210 2480 or +31 70 210 2698

Email: cardioresearch@hagaziekenhuis.nl

Independent Physician: Dr. Vriend, Cardiologist at HagaZiekenhuis

Phone number: +31 70 210 0000

Complaints: Complaints Officer of HagaZiekenhuis

Phone number: +31 70 210 2547 or +31 70 210 1814

Email: klachten.suggesties@hagaziekenhuis.nl

Available by phone from Monday to Friday.

Data Protection Officer of HagaZiekenhuis:

Phone number: +31 70 210 0000 and request to be connected to the Data Protection Officer.

Email: fg@hagaziekenhuis.nl

For more information about your rights: [www.autoriteitpersoonsgegevens.nl](http://www.autoriteitpersoonsgegevens.nl).

**Appendix B: Information about Insurance**

HagaZiekenhuis has taken out insurance for everyone participating in the research. The insurance covers the damages you incur as a result of participating in the research. This includes damages occurring during the research or within 4 years after the research. You must report damages to the insurer within 4 years.

If you have damages due to the research, report it to the following insurer (by phone/email/post):

**Insurer of the research:**

Name: CentraMed

Address: Maria Montessorilaan 9, 2719 DB Zoetermeer

Phone number: +31 70 301 70 70

Email: info@centramed.nl

Policy number: 624.100.025

The insurance covers up to €650,000 per person and €5,000,000 for the entire research.

**Note: The insurance does not cover the following damages:**

- Damages caused by a risk for which information was provided in this letter. This does not apply if the risk turned out to be greater than expected, or if the risk was highly unlikely.
- Damages to your health that would have occurred even if you had not participated in the research.
- Damages resulting from failure to follow instructions or guidance properly.
- Damages to the health of your children or grandchildren.
- Damages caused by an existing treatment method, or by research into an existing treatment method.

These provisions are stated in the "Decision on Compulsory Insurance for Medical-Scientific Research Involving Human Subjects 2015". This decision is available in the Government Gazette (https://wetten.overheid.nl).

**Attachment C: Informed Consent Form**

Related to: Administration of magnesium sulfate to prevent atrial fibrillation around heart surgery.

- I have read the information leaflet. I could also ask questions. My questions have been answered satisfactorily. I had enough time to decide whether to participate.
- I know that participation is voluntary. I also know that I can decide not to participate in the research at any time. Or to stop participating. I do not have to say why I want to stop.
- I give the researcher permission to inform my treating specialist that I am participating in this research.
- I give the researcher permission to request information from my general practitioner/specialist/pharmacist who is treating me about medication I use or about my medical history or complaints.
- I give the researcher permission to provide my general practitioner or specialist with information about unexpected findings from the research that are important for my health.
- I give the researchers permission to collect and use my data. The researchers only do this to answer the research question of this study.
- I know that for the control of the study some people may access all my data. Those people are listed in this information leaflet. I give these people permission to access my data for this control.
- Would you like to check "yes" or "no" in the table below?

I give consent to store my data to use it for other research related to heart arrhythmias, as stated in the information leaflet.

Yes ☐ No ☐

I give consent to be asked if I want to participate in a follow-up study after this research.

Yes ☐ No ☐

I give consent to request my data from another hospital after my surgery (maximum period 28 days after surgery) for the purpose of the current research.

Yes ☐ No ☐

- I want to participate in this research.

My name is (participant): ………………………………..

Signature: ……………………… Date: __ / __ / __

I declare that I have fully informed this participant about the mentioned research.

If any information arises during the study that may affect the participant's consent, I will inform them in a timely manner.

Researcher's name (or representative):……………………………….

Signature:……………………… Date: __ / __ / __

-----------------------------------------------------------------------------------------------------------------

<if applicable>

Additional information provided by:

Name:………………………………..

Position:………………………………

Signature:……………………… Date: __ / __ / __

-----------------------------------------------------------------------------------------------------------------

*The participant will receive a complete information leaflet, along with a signed copy of the consent form.*
