# Supplementary material for: Magnesium sulphate to prevent perioperative atrial fibrillation in cardiac surgery: a randomized clinical trial: A protocol description of the PeriOperative Magnesium Infusion to Prevent Atrial fibrillation Evaluated (POMPAE) trial
Source: Trials. 2024 Aug 15;25:540. doi: 10.1186/s13063-024-08368-3 (PMC11328354; doi:10.1186/s13063-024-08368-3)
Supplement: Supplementary file 3 — Additional file 3: Supplementary Table 2 SPIRIT figure. [file 13063_2024_8368_MOESM3_ESM.docx]

**Supplementary table 2. SPIRIT figure**
